# Gluten Psychosis: Confirmation of a New Clinical Entity

**DOI:** 10.3390/nu7075235

**Published:** 2015-07-08

**Authors:** Elena Lionetti, Salvatore Leonardi, Chiara Franzonello, Margherita Mancardi, Martino Ruggieri, Carlo Catassi

**Affiliations:** 1 Department of Pediatrics, University of Catania, Via S. Sofia 78, 95124 Catania, Italy; E-Mails: leonardi@unict.it (S.L.); franzo.chiara@gmail.com (C.F.); m.ruggieri@unict.it (M.R.); 2 Pediatric Neuro-Psychiatric Unit, G. Gaslini Institute, Via Gerolamo Gaslini 5, 16147 Genova, Italy; E-Mail: margheritamancardi@ospedale-gaslini.ge.it; 3 Department of Pediatrics, Marche Polytechnic University, Ancona, Via Corridoni, 11, 60123 Ancona, Italy; E-Mail: c.catassi@univpm.it; 4 The Division of Paediatric Gastroenterology and Nutrition and Center for Celiac Research, MassGeneral Hospital for Children, 55 Fruit Street, Boston, MA 02114, USA

**Keywords:** gluten, hallucinations, non celiac gluten sensitivity, psycosis

## Abstract

Non-celiac gluten sensitivity (NCGS) is a syndrome diagnosed in patients with symptoms that respond to removal of gluten from the diet, after celiac disease and wheat allergy have been excluded. NCGS has been related to neuro-psychiatric disorders, such as autism, schizophrenia and depression. A singular report of NCGS presenting with hallucinations has been described in an adult patient. We report a pediatric case of a psychotic disorder clearly related to NCGS and investigate the causes by a review of literature. The pathogenesis of neuro-psychiatric manifestations of NCGS is unclear. It has been hypothesized that: (a) a “leaky gut” allows some gluten peptides to cross the intestinal membrane and the blood brain barrier, affecting the endogenous opiate system and neurotransmission; or (b) gluten peptides may set up an innate immune response in the brain similar to that described in the gut mucosa, causing exposure from neuronal cells of a transglutaminase primarily expressed in the brain. The present case-report confirms that psychosis may be a manifestation of NCGS, and may also involve children; the diagnosis is difficult with many cases remaining undiagnosed. Well-designed prospective studies are needed to establish the real role of gluten as a triggering factor in neuro-psychiatric disorders.

## 1. Introduction

Non-celiac gluten sensitivity (NCGS) is a syndrome diagnosed in patients with symptoms that respond to removal of gluten from the diet, after CD and wheat allergy have been excluded [1,2]. The description of this condition is mostly restricted to adults, including a large number of patients previously labeled with “irritable bowel syndrome” or “psychosomatic disorder” [1].

The “classical” presentation of NCGS is, indeed, a combination of gastro-intestinal symptoms including abdominal pain, bloating, bowel habit abnormalities (either diarrhea or constipation), and systemic manifestations including disorders of the neuropsychiatric area such as “foggy mind”, depression, headache, fatigue, and leg or arm numbness [1,2,3]. In recent studies, NCGS has been related to the appearance of neuro-psychiatric disorders, such as autism, schizophrenia and depression [2,4]. The proposed mechanism is a CD-unrelated, primary alteration of the small intestinal barrier (leaky gut) leading to abnormal absorption of gluten peptides that can eventually reach the central nervous system stimulating the brain opioid receptors and/or causing neuro-inflammation. A singular report of NCGS presenting with hallucinations has also been described in an adult patient showing an indisputable correlation between gluten and psychotic symptoms [5].

Here we report a pediatric case of a psychotic disorder clearly related to NCGS.

## 2. Case Report

A 14-year-old girl came to our outpatient clinic for psychotic symptoms that were apparently associated with gluten consumption.

The pediatric ethical committee of the Azienda Universitaria Ospedaliera Policlinico Vittorio Emanuele di Catania approved the access to the patient records. Written informed consent was obtained from the parents of the child.

She was first-born by normal delivery of non-consanguineous parents. Her childhood development and growth were normal. The mother was affected by autoimmune thyroiditis. She had been otherwise well until approximately two years before. In May 2012, after a febrile episode, she became increasingly irritable and reported daily headache and concentration difficulties. One month after, her symptoms worsened presenting with severe headache, sleep problems, and behavior alterations, with several unmotivated crying spells and apathy. Her school performance deteriorated, as reported by her teachers. The mother noted severe halitosis, never suffered before. The patient was referred to a local neuropsychiatric outpatient clinic, where a conversion somatic disorder was diagnosed and a benzodiazepine treatment (*i.e*., bromazepam) was started. In June 2012, during the final school examinations, psychiatric symptoms, occurring sporadically in the previous two months, worsened. Indeed, she began to have complex hallucinations. The types of these hallucinations varied and were reported as indistinguishable from reality. The hallucinations involved vivid scenes either with family members (she heard her sister and her boyfriend having bad discussions) or without (she saw people coming off the television to follow and scare her), and hypnagogic hallucinations when she relaxed on her bed. She also presented weight loss (about 5% of her weight) and gastrointestinal symptoms such as abdominal distension and severe constipation. She was admitted to a psychiatric ward. Detailed physical and neurological examinations, as well as routine blood tests were normal. In order to exclude an organic neuropsychiatric cause of psychosis, the following tests were done: rheumatoid factor, streptococcal antibody tests, autoimmunity profile (including anti-nuclear, anti-double-stranded DNA, anti-neutrophil cytoplasmic, anti-Saccharomyces, anti-phospholipid, anti-mitochondrial, anti-SSA/Ro, anti-SSB/La, anti-transglutaminase IgA (tTG), anti-endomysium (EMA), and anti-gliadin IgA (AGA) antibodies), and screening for infectious and metabolic diseases, but they resulted all within the normal range. The only abnormal parameters were anti-thyroglobulin and thyroperoxidase antibodies (103 IU/mL, and 110 IU/mL; v.n. 0–40 IU/mL). A computed tomography scan of the brain and a blood pressure holter were also performed and resulted normal. Electroencephalogram (EEG) showed mild nonspecific abnormalities and slow-wave activity. Due to the abnormal autoimmune parameters and the recurrence of psychotic symptoms, autoimmune encephalitis was suspected, and steroid treatment was initiated. The steroid led to partial clinical improvement, with persistence of negative symptoms, such as emotional apathy, poverty of speech, social withdrawal and self-neglect. Her mother recalled that she did not return a “normal girl”. In September 2012, shortly after eating pasta, she presented crying spells, relevant confusion, ataxia, severe anxiety and paranoid delirium. Then she was again referred to the psychiatric unit. A relapse of autoimmune encephalitis was suspected and treatment with endovenous steroid and immunoglobulins was started. During the following months, several hospitalizations were done, for recurrence of psychotic symptoms. Cerebral and spinal cord magnetic resonance imaging, lumbar puncture, and fundus oculi examination did not show any pathological signs. Several EEG were performed confirming bilateral slow activity. The laboratory tests showed only mild microcytic anemia with reduced levels of ferritin and a slight increase in fecal calprotectin values (350 mg/dL, normal range: 0–50 mg/dL). In September 2013, she presented with severe abdominal pain, associated with asthenia, slowed speech, depression, distorted and paranoid thinking and suicidal ideation up to a state of pre-coma. The clinical suspicion was moving towards a fluctuating psychotic disorder. Treatment with a second-generation anti-psychotic (*i.e.*, olanzapine) was started, but psychotic symptoms persisted. In November 2013, due to gastro-intestinal symptoms and further weight loss (about 15% of her weight in the last year), a nutritionist was consulted, and a gluten-free diet (GFD) was recommended for symptomatic treatment of the intestinal complaints; unexpectedly, within a week of gluten-free diet, the symptoms (both gastro-intestinal and psychiatric) dramatically improved, and the GFD was continued for four months. Despite her efforts, she occasionally experienced inadvertent gluten exposures, which triggered the recurrence of her psychotic symptoms within about four hours. Symptoms took two to three days to subside again. Then, in April 2014 (two years after the onset of symptoms), she was admitted to our pediatric gastroenterology outpatient for suspected NCGS. Previous examinations excluded a diagnosis of CD because serology for CD was negative (*i.e.*, EMA, and tTG). A wheat allergy was excluded due to negativity of specific IgE to wheat, prick test, prick by prick and patch test for wheat resulted negative. Therefore, we decided to perform a double-blind challenge test with wheat flour and rice flour (one pill containing 4 g of wheat flour or rice flour for the first day, following two pills in the second day and 4 pills from the third day to 15 days, with seven days of wash-out between the two challenges). During the administration of rice flour, symptoms were absent. During the second day of wheat flour intake, the girl presented headache, halitosis, abdominal distension, mood disorders, fatigue, and poor concentration, and three episodes of severe hallucinations. After the challenge, she tested negative for: (1) CD serology (EMA and tTG); (2) food specific IgE; (3) skin prick test to wheat (extract and fresh food); (4) atopy patch test to wheat; and (5) duodenal biopsy. Only serum anti-native gliadine antibodies of IgG class and stool calprotectin were elevated.

Due to parental choice, the girl did not continue assuming gluten and she started a gluten-free diet with a complete regression of all symptoms within a week. The adherence to the GFD was evaluated by a validated questionnaire [6]. One month after AGA IgG and calprotectin resulted negative, as well as the EEG, and ferritin levels improved. She returned to the same neuro-psychiatric specialists that now reported a “normal behavior” and progressively stopped the olanzapine therapy without any problem. Her mother finally recalled that she was returned a “normal girl”. Nine months after definitely starting the GFD, she is still symptoms-free.

## 3. Discussion

To our knowledge, this is the first description of a pre-pubertal child presenting with a severe psychotic manifestation that was clearly related to the ingestion of gluten-containing food and showing complete resolution of symptoms after starting treatment with the gluten-free diet.

Until a few years ago, the spectrum of gluten-related disorders included only CD and wheat allergy, therefore our patient would be turned back home as a “psychotic patient” and receive lifelong treatment with anti-psychotic drugs. Recent data, however, suggested the existence of another form of gluten intolerance, known as NCGS [2,4,7]. NCGS is a condition in which symptoms are triggered by gluten ingestion, in the absence of celiac-specific antibodies and of classical celiac villous atrophy, with variable HLA status and variable presence of first generation AGA. Symptoms usually occur soon after gluten ingestion, disappear with gluten withdrawal and relapse following gluten challenge, within hours or few days. No specific blood test is available for diagnosing NCGS [2].

In our case report, the correlation of psychotic symptoms with gluten ingestion and the following diagnosis of NGCS were well demonstrated; the girl was, indeed, not affected by CD, because she showed neither the typical CD-related autoantibodies (anti-tTG and EMA) nor the signs of intestinal damage at the small intestinal biopsy. Features of an allergic reaction to gluten were lacking as well, as shown by the absence of IgE or T-cell-mediated abnormalities of immune response to wheat proteins. The double-blind gluten challenge, currently considered the gold standard for the diagnosis of NCGS, clearly showed that the elimination and reintroduction of gluten was followed by the disappearance and reappearance of symptoms.

Interestingly, a similar case-report of a 23-years-old female with auditory and visual hallucinations that resolved with gluten elimination has been recently reported [5].

The present case-report confirms that: (a) psychotic disorders may be a manifestation of NCGS; (b) neuro-psychiatric symptoms may involve also children with NCGS; and (c) the diagnosis is difficult and many cases may remain undiagnosed.

The possible causes of psychosis in children and young people are not well understood. It is thought to be the result of a complex interaction of genetic, biological, psychological and social factors. However, we still know relatively little about which specific genes or environmental factors are involved and how these factors interact and actually cause psychotic symptoms [8]. Several studies suggested a relationship between gluten and psychosis [9,10,11,12,13,14,15,16,17,18,19,20,21,22,23,24,25,26,27] or other neuro-psychiatric disorders [28,29,30,31]; however, it remains a highly debated and controversial topic that requires well-designed prospective studies to establish the real role of gluten as a triggering factor in these diseases [2,27].

On the other hand, the pathogenesis of neuro-psychiatric manifestations of NCGS is an intriguing and still poorly understood issue. It has been hypothesized that some neuro-psychiatric symptoms related to gluten may be the consequence of the excessive absorption of peptides with opioid activity that formed from incomplete breakdown of gluten. Increased intestinal permeability, also referred to as “leaky gut syndrome”, may allows these peptides to cross the intestinal membrane, enter the bloodstream, and cross the blood brain barrier, affecting the endogenous opiate system and neurotransmission within the nervous system [2,32]. Interestingly, in our case, we observed an elevation of fecal calprotectin that resolved during gluten-free diet, suggesting that a certain degree of gut inflammation may be found in NCGS. The role of stool calprotectin as a biomarker of NCGS requires further evaluation.

Recently, a higher prevalence of antibodies directed toward tTG6 (a transglutaminase primarily expressed in the brain) has been observed in adult patients affected by schizophrenia [26]; this finding suggests that these autoantibodies could have a role in the pathogenesis of the neuro-psychiatric manifestations seen in NCGS. It is possible that gluten peptides (either directly or through activation of macrophages/dendritic cells) may set up an innate immune response in the brain similar to that described in the gut mucosa, causing exposure of tTG6 from neuronal cells. Access of these gluten peptides and/or activated immune cells to the brain may be facilitated by a breach of the blood brain barrier [26]. Evidence from the literature supports the notion that a subgroup of psychotic patients shows increased expression of inflammatory markers including haptoglobin-2 chains α and β [33]. Zonulin is a tight junction modulator that is released by the small intestine mucosa upon gluten stimulation. Interestingly the zonulin receptor, identified as the precursor for haptoglobin-2, has been found in the human brain. Overexpression of zonulin (aka haptoglobin-2) could be involved in the blood brain barrier disruption similarly to the role that zonulin plays in increasing intestinal permeability. This hypothesis is supported by the observation that zonulin analogues can modulate the blood brain barrier by increasing its permability to high molecular weight markers and chemotherapeutic agents. In recent years, there has been a growing emphasis on early detection and intervention of psychotic symptoms in order to delay or possibly prevent the onset of psychosis and schizophrenia [8]. Children and young people with schizophrenia tend to have a shorter life expectancy than the general population, largely because of suicide, injury, or cardiovascular disease, the last partly related to chronic treatment with antipsychotic medication [34]. Moreover, psychotic disorders in children and young people (up to age 17 years) are the leading causes of disability, owing to disruption to social and cognitive development [8]. Shedding light on the possible role of gluten in this context may significantly change the life for a subset of these patients, as shown by the case described in this case-report.

## 4. Conclusions

The present case report shows that psychosis may be a manifestation of NCGS, and may also involve children; the diagnosis is difficult with many cases remaining undiagnosed. The pathogenesis of neuropsychiatric manifestations of NCGS is an intriguing and still poorly understood issue. Well designed prospective studies are needed to establish the real role of gluten as a triggering factor in these diseases.
